# Aging the death: the importance of having better methods for age at death estimation of old individuals

**DOI:** 10.1080/07853890.2021.1893522

**Published:** 2021-09-28

**Authors:** Eugénia Cunha

**Affiliations:** a National Institute of Legal Medicine and Forensic Sciences, Lisbon, Portugal; b Laboratory of Forensic Anthropology, University of Coimbra, Coimbra, Portugal

## Abstract

While trying to return the identity to human remains, the forensic anthropologist has to estimate four basic parameters: sex, age at death, ancestry, and stature. These are the so-called big four parameters of identification which altogether with the identity factors can allow a positive identification, which enables the return of the remains to the families. This presentation focusses the problematic of age estimation of older individuals which is a very relevant question since a lot of "John Doe's" are old individuals who lived alone and/or who got lost. Besides, with the increase in life expectancy, this is, more and more, a significant concern. The issue is then a different forensic perspective of aging. Until very recently, all we could do was to tell that the individual was older than 60 years when he/she died, which was not very helpful to narrow down the possibilities, since after that age, with the increasing longevity, we can have individuals dying at seventies, eighties, nineties or even centenarians. Recent research has been permitting to do some discrimination among these older age groups. However, more research about how the skeletal system ages, a paramount question, is needed. While growth and development are programmed strictly by evolution and genetics, the same does not apply to adult degeneration process. Hence, not all old individuals have old skeletons.

All the approaches to adult age estimation only produce estimates of age (age groups) with a relatively wide age range, and, the older the individuals, the wider is that age range. The perspectives and limitations of aging the older are discussed. It is clear that no single technique is able to provide an accurate estimation, the key is to use multiple methods and, above all, on how to combine them. This last issue has been profiting from the application of updated and appropriate mathematical techniques. Regarding skeletal age indicators, it is now possible to tell which should not be used. The ones to be analysed are also dictated by the state of preservation and completeness of the skeletal remains. Some of these skeletal age indicators will be commented and the contribution of genetics to this issue will also be approached.

The relevance of this subject will be illustrated through the presentation of some practical forensic cases.

